# Cell Adhesion Motif-Functionalized Lipopeptides: Nanostructure and Selective Myoblast Cytocompatibility

**DOI:** 10.1021/acs.biomac.2c01068

**Published:** 2022-12-15

**Authors:** Elisabetta Rosa, Lucas de Mello, Valeria Castelletto, Mark L. Dallas, Antonella Accardo, Jani Seitsonen, Ian W. Hamley

**Affiliations:** † School of Chemistry, Pharmacy and Food Biosciences, University of Reading, Whiteknights, Reading, Berkshire RG6 6AD, U.K.; ‡ Department of Pharmacy and Research Centre on Bioactive Peptides (CIRPeB), University of Naples “Federico II”, Via Domenico Montesano 49, Naples 80131, Italy; § Departamento de Biofísica, Universidade Federal de São Paulo, São Paulo 04023-062, Brazil; ∥ Nanomicroscopy Center, Aalto University, Puumiehenkuja 2, Espoo FIN-02150, Finland

## Abstract

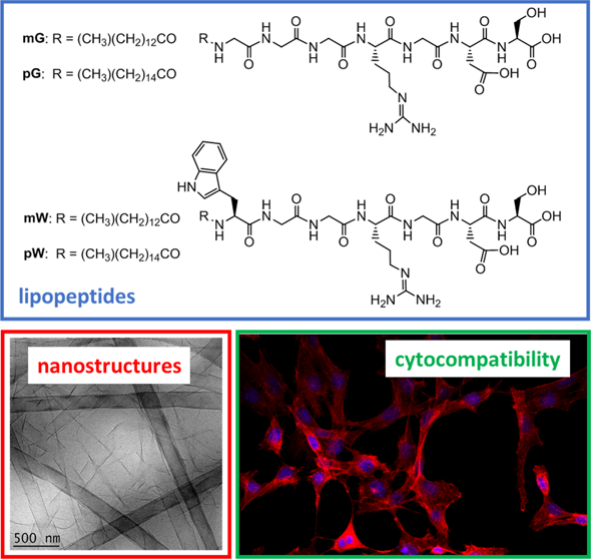

The conformation and self-assembly of four lipopeptides, peptide amphiphiles comprising peptides conjugated to lipid chains, in aqueous solution have been examined. The peptide sequence in all four lipopeptides contains the integrin cell adhesion RGDS motif, and the cytocompatibility of the lipopeptides is also analyzed. Lipopeptides have either tetradecyl (C_14_, myristyl) or hexadecyl (C_16_, palmitoyl) lipid chains and peptide sequence WGGRGDS or GGGRGDS, that is, with either a tryptophan-containing WGG or triglycine GGG tripeptide spacer between the bioactive peptide motif and the alkyl chain. All four lipopeptides self-assemble above a critical aggregation concentration (CAC), determined through several comparative methods using circular dichroism (CD) and fluorescence. Spectroscopic methods [CD and Fourier transform infrared (FTIR) spectroscopy] show the presence of β-sheet structures, consistent with the extended nanotape, helical ribbon, and nanotube structures observed by cryogenic transmission electron microscopy (cryo-TEM). The high-quality cryo-TEM images clearly show the coexistence of helically twisted ribbon and nanotube structures for C_14_-WGGRGDS, which highlight the mechanism of nanotube formation by the closure of the ribbons. Small-angle X-ray scattering shows that the nanotapes comprise highly interdigitated peptide bilayers, which are also present in the walls of the nanotubes. Hydrogel formation was observed at sufficiently high concentrations or could be induced by a heat/cool protocol at lower concentrations. Birefringence due to nematic phase formation was observed for several of the lipopeptides, along with spontaneous flow alignment of the lyotropic liquid crystal structure in capillaries. Cell viability assays were performed using both L929 fibroblasts and C2C12 myoblasts to examine the potential uses of the lipopeptides in tissue engineering, with a specific focus on application to cultured (lab-grown) meat, based on myoblast cytocompatibility. Indeed, significantly higher cytocompatibility of myoblasts was observed for all four lipopeptides compared to that for fibroblasts, in particular at a lipopeptide concentration below the CAC. Cytocompatibility could also be improved using hydrogels as cell supports for fibroblasts or myoblasts. Our work highlights that precision control of peptide sequences using bulky aromatic residues within “linker sequences” along with alkyl chain selection can be used to tune the self-assembled nanostructure. In addition, the RGDS-based lipopeptides show promise as materials for tissue engineering, especially those of muscle precursor cells.

## Introduction

The integrin cell adhesion peptides RGD and RGDS are minimal units of a domain present in proteins such as fibrinogen, fibronectin, and vitronectin.^1,2^ These sequences have been extensively used in the development of synthetic bionanomaterials for applications in cell growth and differentiation or tissue scaffolding^3−12^ and for the delivery of therapeutics.^13−15^ The RGDS tetrapeptide has antithrombolytic activity resulting from the inhibition of platelet aggregation due to the fibrinogen recognition sequence.^16,17^ The RGD and RGDS motifs have been incorporated into lipopeptides, one type of peptide amphiphile (PA).^6,9,18−30^ In some reports, the RGDS tetrapeptide is purported to have enhanced bioactivity compared to RGD due to the additional serine residue.^31−35^ The self-assembly of materials containing RGD-peptide sequences has been reviewed elsewhere.^11,35^

Lipopeptides comprise a peptide sequence attached to one or more alkyl chains. This confers amphiphilicity and leads to self-assembly, often into extended fibril structures, although other morphologies can be accessed through appropriate molecular design. The remarkable self-assembly properties of lipopeptides have been reviewed elsewhere.^36−43^ Lipidation is also a practical strategy used to enhance the stability of peptide therapeutics in vivo,^44,45^ and extended fibril structures with a lipid core enable the presentation of peptide motifs at high density on the surface, potentially enhancing bioactivity.^40,42,46^

Lipopeptides self-assemble in solution into a range of nanostructures of which elongated nanostructures (fibrils and nanotapes) are the most commonly reported, although micelles and vesicles have also been observed in a few cases.^36−43^ Remarkably, some lipopeptides form nanotubes in which the nanotube wall comprises molecular bilayers (i.e., with a peptide coating on both the inner and outer surface). This mode of self-assembly results from the closure of helical ribbon structures arising from the twisting of lipopeptide nanotapes.^47−50^ This has also been observed for certain unlipidated amyloid peptides.^48,49,51−63^ Other types of peptide nanotubes can also form via distinct mechanisms including the direct packing of cyclic peptides,^64,65^ from coiled-coil oligomerization of parallel arrays of α-helical peptides into hollow-core aggregates,^66−68^ from packing of α-helices perpendicular to the nanotube wall in a so-called “cross-α” nanotube architecture,^69−71^ or others.^72−78^ The mode of self-assembly of lipopeptides depends on a variety of molecular features including peptide sequence (charge and its distribution, placement of bulky residues, etc.) and lipid chain length. This is exploited herein, in a comparison of four lipopeptides with two different peptide sequences (but retaining the same bioactive RGDS motif) and two different lipid chains, both of sufficient length (above C_12_^79^) to lead to amphiphilicity and hence self-assembly. Due to the remarkable range of self-assembled structures of lipopeptides and their diversity of activities, there is great interest in the use of these bioinspired/bioderived materials for applications in nanotechnology, nanobiotechnology, and nanomedicine.

**Scheme 1:**
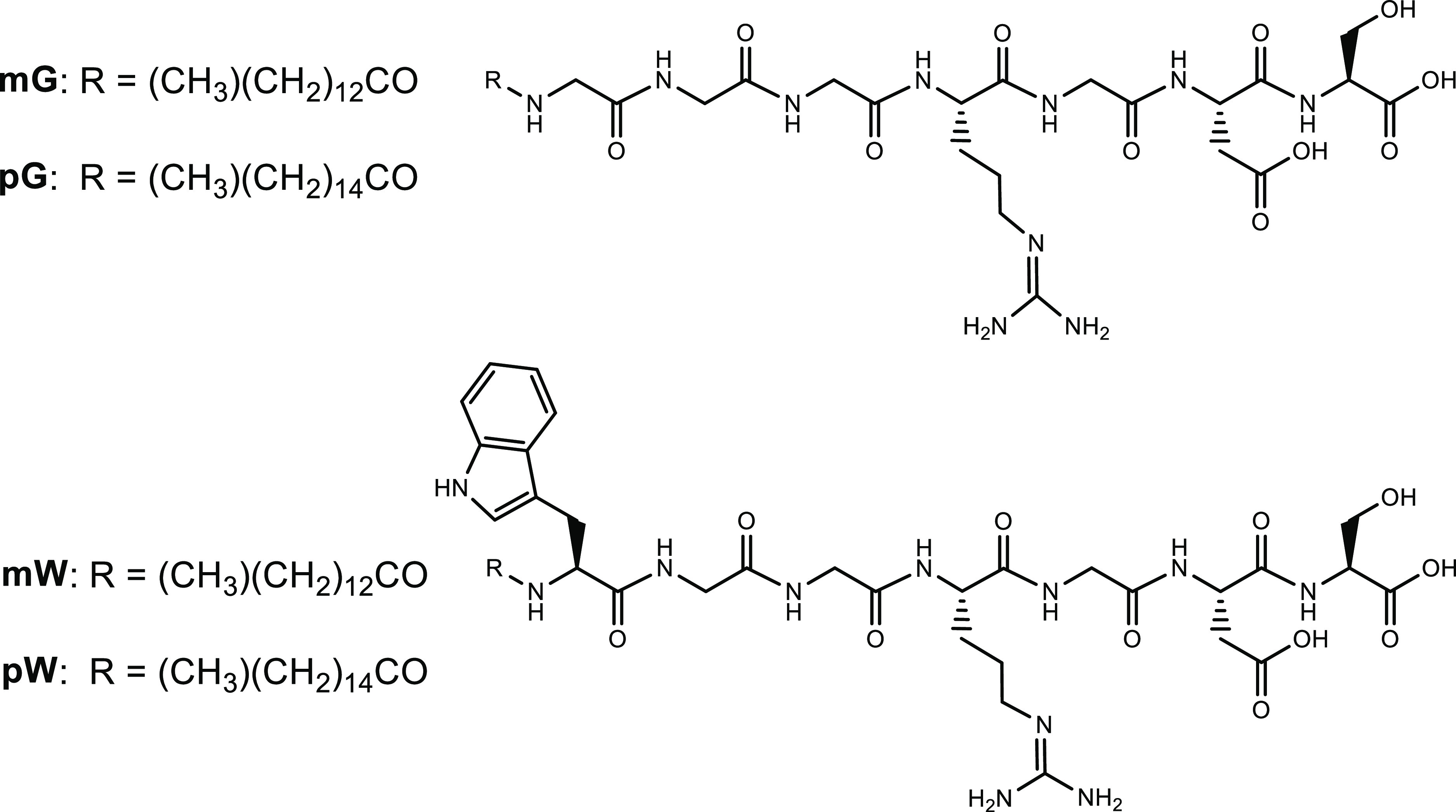
Structures of Lipopeptides Studied

Here, we investigate the effect of the sequence and alkyl chain length on the self-assembly and cytocompatibility of lipopeptides bearing a bioactive C-terminal RGDS (arginine–glycine–aspartic acid–serine) cell adhesion motif sequence. The RGDS sequence is linked to the alkyl chain via a spacer, either GGG or WGG; that is, the N-terminal residue comprises either just the simple (nonchiral) glycine (G) or with one G replaced with bulky aromatic tryptophan (W). In the latter case, tryptophan can also serve as a fluorescence reporter. We also compare the behaviors of lipopeptides bearing myristyl (tetradecyl, C_14_) or palmitoyl (hexadecyl, C_16_) lipid chains. The structures of the lipopeptides are shown in Scheme 1. The Hamley group previously studied the self-assembly of C_16_-GGGRGDS (pG) in comparison with C_16_-GGGRGD,^24^ and their mixtures with C_16_-ETTES, the latter serving as a negatively charged diluent in our development of supports for tissue engineering.^80^ This work has subsequently led to the development of bioactuators for corneal tissue engineering (curved cornea-shaped stromal tissue equivalents)^28^ and RGD-terminated lipopeptides which also incorporate matrix metalloprotease substrate sequences have also been used in the development of protease-responsive self-releasing tissue as part of a project to create a biomimetic cornea.^81,82^ Lipopeptides C_16_-GGGRGDS and C_16_-GGGRGD both self-assemble into nanotapes with an internal bilayer structure.^24^ We also investigated the interaction of C_16_-GGGRGDS with apolipoprotein-AI.^83^

Here, we find that self-assembly can be tuned by the incorporation of a bulky residue (tryptophan) and/or by adjustment of the lipid chain length. We also carefully examined the cytocompatibility of the lipopeptides comparing the fibroblast and myoblast cell lines. Although both cell types are used in tissue engineering and regenerative medicine, myoblasts are precursors of muscle cells and are of particular current interest in the Hamley laboratory for a project on new biomaterials for cultured (synthetic) meat. The production of cultured meat involves the in vitro generation of muscle cells from myoblasts.^84−87^ Tissue engineering of skeletal muscle or cardiac muscle also relies on myoblast cytocompatibility.^88−92^ Previous work has demonstrated the use of lipopeptides in tissue engineering of smooth muscle cells with enhanced cytocompatibility and/or bioactivity,^93−99^ although, to the best of our knowledge, the compatibility of lipopeptides with myoblasts has not been demonstrated. This is of interest since the use of myoblasts may be advantageous in controlling the differentiation process and ultimate cell morphology.

## Experimental Section

### Materials

Peptides were obtained from Peptide Synthetics (Peptide Protein Research), Farnham, UK, as trifluoroacetic acid (TFA) salts with >95% purity as confirmed by reverse phase-high-performance liquid chromatography (RP-HPLC). Molar masses by electrospray ionization mass spectrometry are 814.94 g mol^–1^ (C_14_-GGGRGDS, mG), 842.98 g mol^–1^ (C_16_-GGGRGDS, pG), 943.68 g mol^–1^ (C_14_-WGGRGDS, mW), and 971.72 g mol^–1^ (C_16_-WGGRGDS, pW).

### Sample Preparation

All the peptide solutions were prepared by dissolving the peptides at different concentrations in a phosphate-buffered saline (PBS) solution at 10 mmol L^–1^ at pH = 7.4.

### CD Spectroscopy

Far-UV circular dichroism (CD) spectra were collected using a Chirascan spectropolarimeter (Applied Photophysics, Leatherhead, UK) equipped with a thermal controller. Peptides solved in PBS were placed in 0.1 or 10 mm quartz cells depending on their concentration (5 × 10^–5^, 1 × 10^–4^, 5 × 10^–4^, 1 × 10^–3^, 5 × 10^–3^, 1 × 10^–2^, 5 × 10^–2^, 1 × 10^–1^, and 5 × 10^–1^ and 1 wt %). Spectra were recorded from 280 to 195 nm. Other experimental settings were 0.5 nm step, 1 nm bandwidth, and 1 s collection time per step. Each spectrum was obtained by averaging three scans and correcting for the blank. The critical aggregation concentrations (CACs) of the peptides were obtained by plotting the molar ellipticity at the wavelength of maximum ellipticity, λ_max_, as a function of the concentration.

### Fourier Transform Infrared (FTIR) Spectroscopy

A Thermo-Scientific Nicolet iS5 instrument equipped with a DTGS detector, with a Specac Pearl liquid cell with CaF_2_ plates to fix the sample, was used to collect the spectra of peptides at the concentration of 0.1, 0.5, and 1 wt % for mG and pG and 0.1 and 0.5 wt % for mW and pW. A total of 128 scans for each sample were recorded over the range of 900–4000 cm^–1^.

### Polarized Optical Microscopy

A drop of the peptide solution was placed on a microscope slide and stained with a 1 wt % Congo red aqueous solution. Congo red is a dye which shows apple-green birefringence when staining amyloid β-sheet structures.^100−102^ After covering the sample with a microscope coverslip, it was observed through the crossed polarizers of an Olympus BX41 polarized microscope. Images were captured using a Canon G2 digital camera fitted to the microscope.

### Fluorescence Spectroscopy

Fluorescence experiments were carried out using a 10.0 mm × 5.0 mm quartz cell in a Varian Model Cary Eclipse spectrofluorometer. Excitation and emission bandwidths of 2.5 nm were used as experimental settings. The temperature was set at 20 °C for all the experiments.

The CAC for all the peptides was assessed by fluorescence experiments with thioflavin T (ThT), a cationic benzothiazole dye that shows enhanced fluorescence around 485 nm upon binding to amyloid fibers.^101−104^ Peptide solutions at different concentrations were prepared by dissolving the peptide powder in a PBS solution of ThT, concentration 50 μmmol L^–1^. Samples were excited at 450 nm, and fluorescence emission spectra were recorded between 460 and 600 nm.

Emission spectra for mW and pW at different concentrations were collected from 290 to 500 nm after excitation at 280 nm. The self-fluorescence properties were studied by exciting the peptides at a range of wavelengths between 350 and 580 nm.

### Small-Angle X-ray Scattering

Small-angle X-ray scattering experiments were performed on beamline SWING at the SOLEIL synchrotron (Gif-sur-Yvette, France).^105^ Solution samples were delivered to a quartz capillary under vacuum in the X-ray beam using a BioSAXS setup. Gels were loaded into a plastic support sandwiched between two polyimide foils held in place by a metal frame. Data were collected using an in-vacuum EigerX-4M detector, with an X-ray wavelength of 1.033 Å at two sample-to-detector distances, 6.217 and 0.517 m. Data were reduced to one-dimensional form (except the raw two-dimensional patterns where anisotropy was observed) and averaged and background-subtracted using the software Foxtrot.^105^

### Cryogenic-TEM

Imaging was carried out using a field emission cryoelectron microscope (JEOL JEM-3200FSC), operating at 200 kV. Images were taken in bright field mode and using zero loss energy filtering (omega type) with a slit width of 20 eV. Micrographs were recorded using a Gatan Ultrascan 4000 CCD camera. The specimen temperature was maintained at −187 °C during the imaging. Vitrified specimens were prepared using an automated FEI Vitrobot device using Quantifoil 3.5/1 holey carbon copper grids with a hole size of 3.5 μm. Just prior to use, grids were plasma-cleaned using a Gatan Solarus 9500 plasma cleaner and then transferred into the environmental chamber of a FEI Vitrobot at room temperature and 100% humidity. Thereafter, 3 μL of the sample solution was applied on the grid and it was blotted twice for 5 s and then vitrified in a 1/1 mixture of liquid ethane and propane at a temperature of −180 °C. The grids with the vitrified sample solution were maintained at liquid nitrogen temperature and then cryotransferred to the microscope.

### Cell Lines

L929 murine fibroblast and C2C12 immortalized mouse myoblast cell lines (both from ECACC General Cell Collection) were grown in Dulbecco’s modified Eagle’s medium (DMEM) supplemented with 10% fetal bovine serum, 20 mM HEPES, and 1% GlutaMAX. The cells were maintained at pH 7.4, 37 °C, and 5% CO_2_ in 25 cm^2^ cell culture flasks.

### Cytotoxicity Assays

Cells were seeded in 96-well plates at a density of 0.6 × 10^4^ cells per well. Cells were then treated with peptides dissolved in the medium at the concentrations of 0.1 and 1 × 10^–4^ wt %. To test cytocompatibility in the presence of the hydrogels, wells were filled with 1 wt % mG and pG hydrogels before seeding. At the end of the treatment (after 24 or 72 h), cell viability was assessed using an MTT [3-(4, 5-dimethylthiazolyl-2)-2, 5-diphenyltetrazolium bromide] assay. In brief, after the removal of the culture medium, MTT, dissolved in DMEM at a concentration of 0.5 mg/mL, was added to the cells and incubated for 4 h at 37 °C. The resulting formazan crystals were dissolved by adding dimethyl sulfoxide. Absorbance values of blue formazan were determined at 560 nm using an automatic plate reader. Cell survival was expressed as a percentage of viable cells in the presence of peptides, compared to control cells grown in their absence. The assay was repeated three times, and the results were averaged. Statistical significance was tested using multiple Welch’s *t*-tests. All analyses were conducted using Prism 7.

## Results and Discussion

### Secondary Structure and CAC

**Figure 1:**
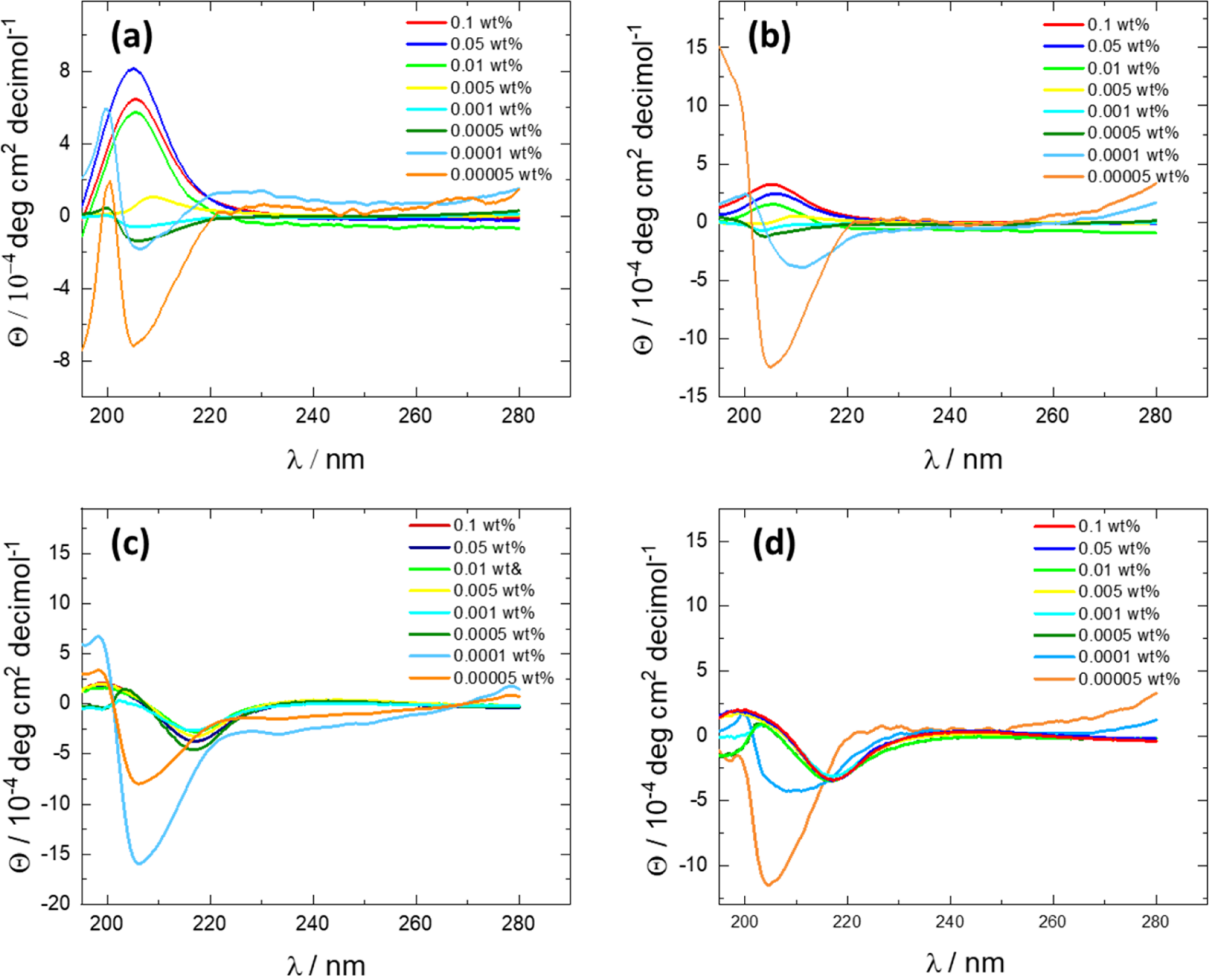
CD spectra at the concentrations indicated for (a) mG, (b) pG, (c) mW, and (d) pW.

The secondary structure of the four lipopeptides was probed in PBS solutions using CD and FTIR spectroscopy. At lower concentrations, all the peptides present a random coil organization, confirmed by the shape of the CD spectra (Figure 1), characterized by a minimum at 205 nm. At the highest concentrations, for mG and pG, the CD spectra are dominated by a positive band with a maximum centered at 205 nm, while for mW and pW spectra, a characteristic of β-sheet structures is observed,^106−108^ with a negative band centered at 216 nm and a positive band centered at 203 nm. The CD spectra of elongated PA nanostructures typically present positive bands due to β-sheets at ∼200–205 nm and negative ones around 220 nm, associated with π → π* and *n* → π* transitions, respectively. However, it is possible to find some cases in which the signal around 220 nm is only weakly or not detectable in non-aromatic PAs.^109^ The lack of the typical negative CD band can be due to overlap with the absorption region of other groups such as arginine, as previously observed for PAs containing the RGD sequence.^24^ Although mG and pG do not show typical β-sheet CD profiles above the CAC (they lack a minimum near 216 nm), the presence of β-sheet structures was confirmed by FTIR spectroscopy to be discussed shortly, as well as the observation of extended nanostructures by cryogenic-TEM (cryo-TEM) and small-angle X-ray scattering (SAXS) (vide infra).

**Figure 2:**
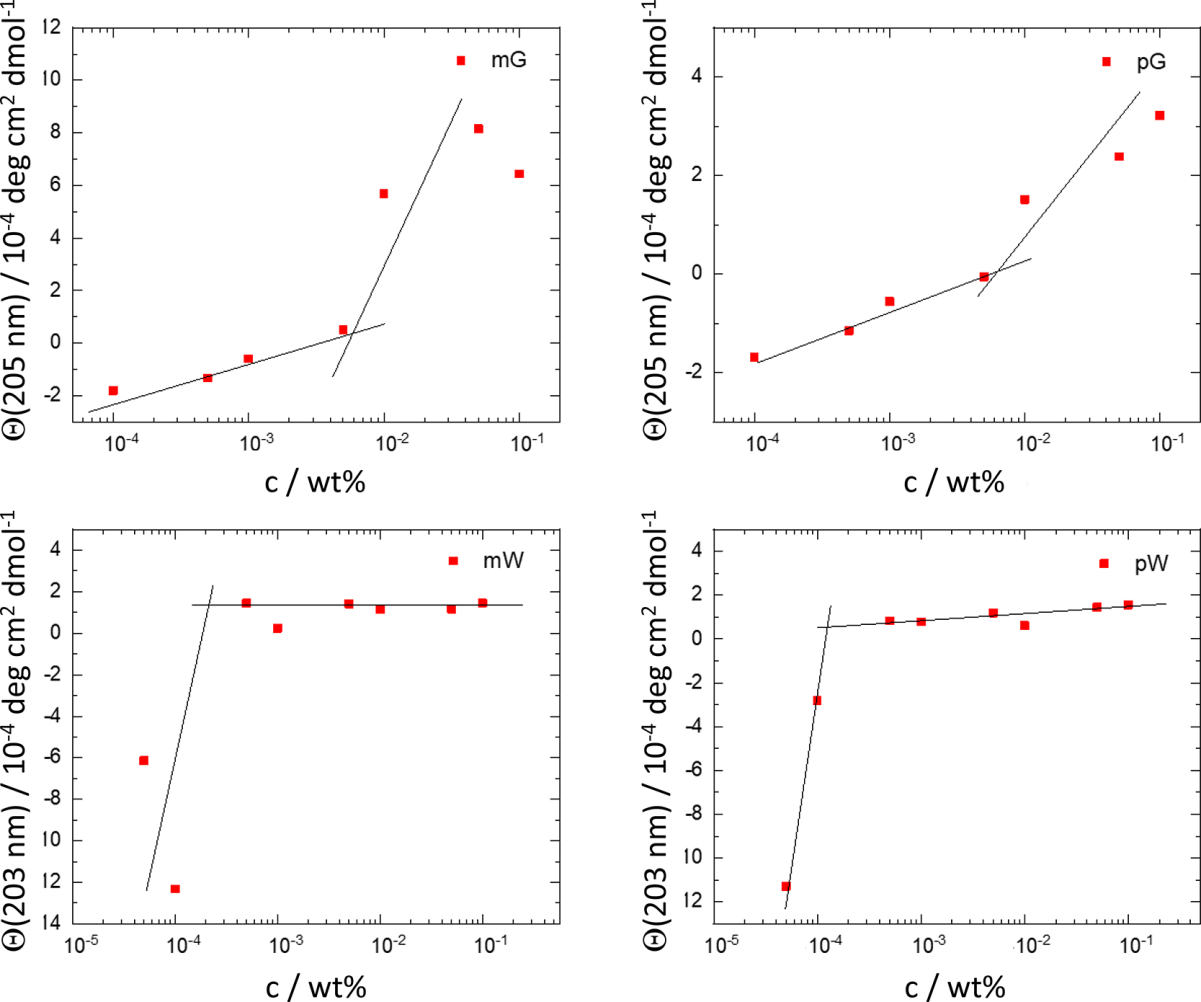
Determination of CAC values from concentration-dependent discontinuities in molar ellipticity values (at 205 nm for mG and pG and 203 nm for mW and pW).

To check the consistency of different methods of determining CAC values, we compared data from previously employed fluorescence probes and tryptophan fluorescence methods with less widely used analysis of discontinuities in CD spectra. The former are sensitive to changes in the local environment of the fluorophore upon aggregation (e.g., the formation of hydrophobic domains), whereas the latter is sensitive to the secondary structure of the peptide, which may also change in the self-assembled state. It should also be noted that analysis of CD spectra or W fluorescence avoids the use of added probe molecules, which could potentially influence aggregation properties. Considering CD first, the CACs were initially estimated by plotting the molar ellipticity of the first positive maximum near 205 nm as a function of the concentration. The data is shown in Figure 2. The CACs determined from the intersection point of linear extrapolations of the data were found to be (6.02 ± 0.03) × 10^–3^ wt %, (6.49 ± 0.08) × 10^–3^ wt %, (2.16 ± 0.05) × 10^–4^ wt %, and (1.26 ± 0.03) × 10^–4^ wt % for mG, pG, mW, and pW, respectively.

For comparison, the CAC was also obtained for the two tryptophan-containing lipopeptides from W self-fluorescence measurements. Emission spectra of the two tryptophan-containing peptides were collected at different concentrations after excitation at 280 nm. At higher concentrations, an emission peak at 320 nm is visible. This shift indicates that tryptophan is located in a hydrophobic environment.^110−112^ By plotting the fluorescence intensity at 320 nm as a function of the concentration (Figure S1), the concentration at the break point (corresponding to the CAC) was found to be (2.16 ± 0.05) × 10^–4^ wt % for mW and (3.75 ± 0.04) × 10^–4^ wt % for pW. These data are consistent with those obtained with the CD studies. For all the peptides, the CAC was also determined by collecting emission spectra of the amyloid-sensitive dye ThT in the presence of increasing amounts of the lipopeptide. Plotting the fluorescence intensity at 482 nm as a function of the concentration (Figure S2) leads to CAC values of (4.59 ± 0.05) × 10^–3^ wt %, (2.87 ± 0.04) × 10^–3^ wt %, (3.10 ± 0.07) × 10^–3^ wt %, and (3.55 ± 0.08) × 10^–3^ wt % for mG, pG, mW, and pW, respectively. The values for mG and pG are in good agreement with those obtained from the analysis of CD spectra; however, the values for mW and pW are significantly (by about an order of magnitude) higher than those from CD or tryptophan fluorescence. This is ascribed to interference between ThT and W fluorescence (e.g., resonance energy transfer), and also, it is well documented that W in its aggregated form can exhibit a weak emission that can overlap with the spectral region of ThT-aggregates (450–480 nm). These findings suggest that measurements using ThT fluorescence in the presence of peptides containing tryptophan should be used with caution. The CAC values obtained from the different methods are compared in Table S2.

The value of the CAC for pG may be compared to the previously reported CAC value determined from ThT fluorescence, CAC = 4.7 × 10^–3^ wt %.^24^ The value reported here is reassuringly consistent with that obtained from a separate measurement on a different batch of the sample, and we also highlight the consistent value obtained from ThT and CD for mG and pG.

**Figure 3:**
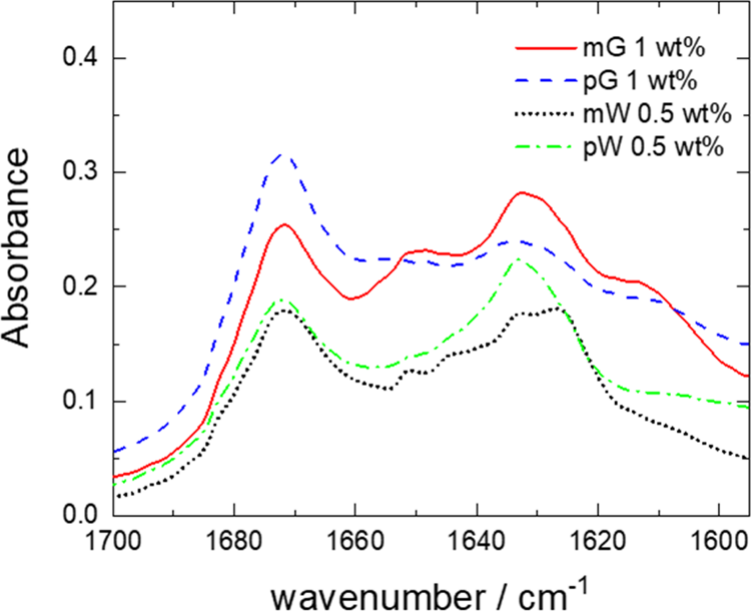
FTIR spectra at the concentrations indicated.

Further information on the secondary structure of self-assembled peptides was obtained from FTIR spectroscopy. FTIR spectra were measured for 0.1 and 1 wt % mG and pG and 0.1 and 0.5 wt % mW and pW. Spectra are plotted in the region of the amide I region, from 1700 and 1600 cm^–1^, as this is used to characterize the secondary structure of peptides.^45,101,113−116^ The FTIR spectra shown in Figures 3 and S3 for the four lipopeptides suggest that solutions of all of them contain a significant β-sheet structure because of the presence of bands at 1631 (and 1612 cm^–1^) for mG and pG and 1633 and 1626 cm^–1^ for mW and pW.^45,101,113,115,116^ The peak at 1672 cm^–1^ is attributed to the TFA counterions bound to cationic residues in the peptide^114,117,118^ (here: arginine). A minor α-helix component can be observed for all the peptides, as shown by the band centered at 1651 cm^–1^, while the spectra for mG and pG also contain a minor random coil component, which gives rise to the peak at 1644 cm^–1^. The presence of β-sheet peaks in the FTIR spectra for mG and pG indicates that this structure forms at a higher concentration (1 wt % used for FTIR measurements) than those studied by CD (Figure 1a,b).

It is known that β-sheet aggregates can undergo self-fluorescence.^119−121^ Self-fluorescence of the two myristoyl-modified peptides (mW and mG) at a concentration of 0.05 wt % was tested by exciting the sample in a range of wavelengths between 350 and 580 nm. A self-fluorescence phenomenon is visible after excitation between 380 and 400 nm (Figure S4).

### Self-Assembly and Gelation

**Figure 4:**
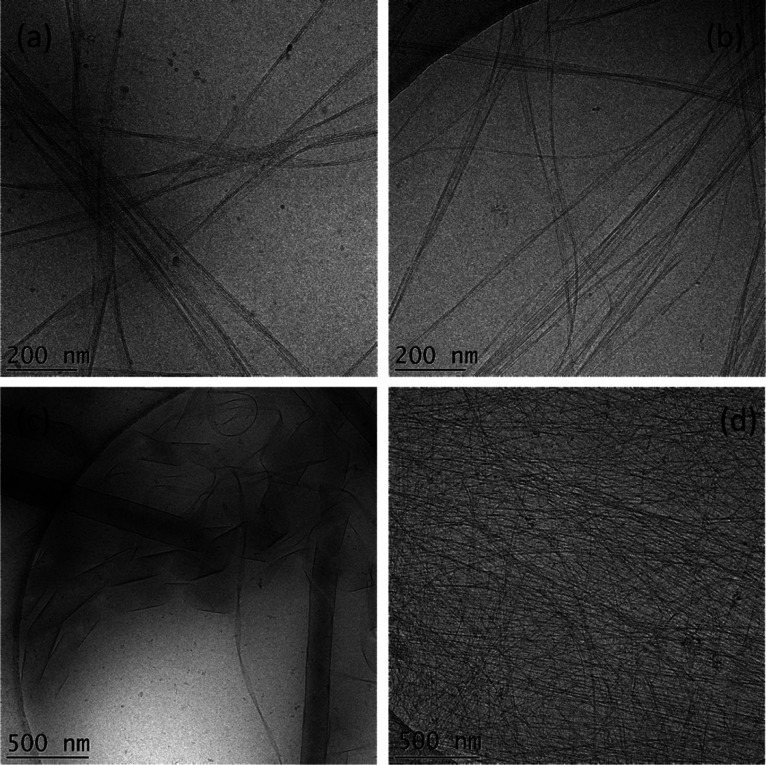
Cryo-TEM images from 0.1 wt % solutions (a) mG, (b) pG, (c) mW, and (d) pW.

Cryo-TEM was used to image self-assembled nanostructures in aqueous solution. The images for 0.1 wt % solutions are shown in Figure 4 (additional selected images are presented in Figure S5) and show that mG forms twisted nanotape structures which notably comprise arrays of individual filaments. A similar structure is observed for pG, although with a less pronounced filament structure within the nanotapes. The nanostructures in a solution of mW and pW are distinct from those of the two glycine-containing lipopeptides. Remarkably, mW forms right-handed twisted helical ribbons coexisting with closed nanotubes (in which the wrapped helical ribbon structure can still be seen within the walls) (Figure 4c). The mean diameter of the nanotubes is (178 ± 25) nm with a wall thickness of less than 10 nm. The coexistence of helical ribbons and nanotubes provides visual evidence for the mechanism of nanotube formation via the closure of helical ribbon structures. This process has been reported previously for several lipopeptide systems^47,48,57,60,122,123^ and shows that nanotube walls comprise layers of lipopeptide molecules arranged perpendicular to the tube walls. The Cryo-TEM images for the sample pW (e.g., Figure 4d) show that it forms a dense network of long intertwined thin fibers with a mean diameter of 4.0 nm. The cryo-TEM images show that the presence of tryptophan in mW and pW significantly alters the molecular packing compared to nonchiral and flexible glycine in mG and pG, and this in turn influences the nanostructure.

**Figure 5:**
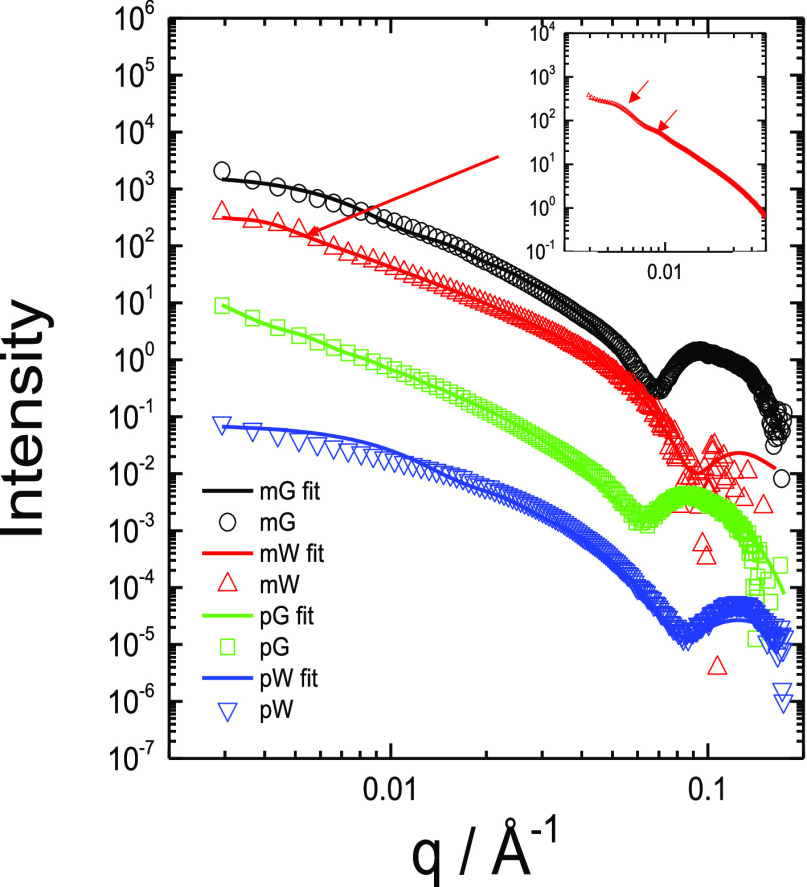
SAXS data from solutions (every fifth data point shown and curves shifted for ease of visualization, along with the model form factor fits described in the text). Inset: data for mW at low *q* on an expanded intensity scale (and with all data points shown along with form factor fit) to show form factor oscillations resulting from the nanotube structure. The solution concentrations and form factor fit parameters are listed in Table S1.

Cryo-TEM was complemented with SAXS which provides quantitative information on the shape and dimensions of self-assembled nanostructures via analysis of the form factor.^124^ SAXS data for solutions of the four lipopeptides is shown in Figure 5. Consistent with cryo-TEM, the form factors for mG, pG, and pW can be fitted using form factors of nanotapes with an internal bilayer structure (hydrophobic lipid and peptide sublayers). The fit parameters are listed in Table S1. The bilayer thickness is in the range of 35–46 Å. Considering the estimated molecular lengths for myristyl-conjugated heptapeptides (42 Å) or palmitoyl-conjugated heptapeptides (44 Å), these values indicate highly interdigitated bilayers and/or regions where residues are not in extended β-sheet conformation. As revealed by cryo-TEM, mW exhibits unique self-assembly behavior into helical ribbons coexisting with nanotubes. The SAXS form factor data could be fitted using a simple model of nanotubes (i.e., a cylindrical shell), as shown in Figure 5. The fit parameters in Table S1 indicate a nanotube radius of 750 Å, consistent with the cryo-TEM image in Figure 4c. The nanotube wall thickness is 64 Å; that is, it comprises a bilayer (of partly interdigitated molecules), this also being consistent with cryo-TEM.

**Figure 6:**
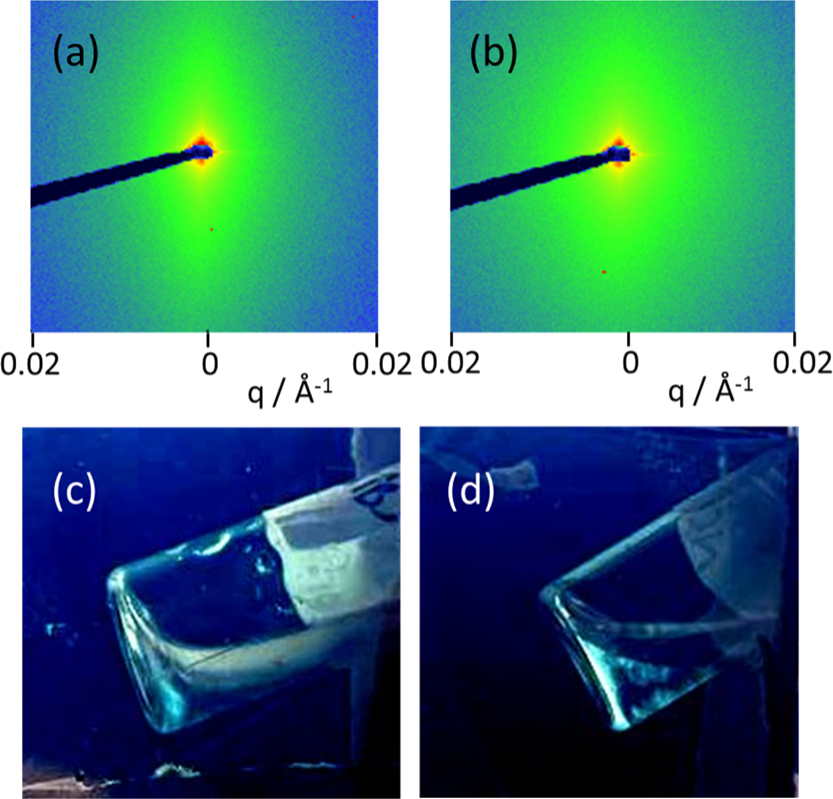
Data showing nematic phase formation by mW and pW (0.5 wt % solutions). Spontaneous alignment in SAXS patterns due to flow in capillaries: (a) mW and (b) pW. Images of solutions in vials between crossed polarizers: (c) mW and (d) pW.

Two-dimensional SAXS patterns (Figure 6a,b) show that mW and pW solutions (0.5 wt %) show strong anisotropy at low wavenumber *q*, indicating that the samples comprise nematic phases which align under flow (some anisotropy was also observed for the corresponding solution of mG). This feature was also confirmed by the macroscopic birefringence examined for samples placed in glass vials between crossed polarizers (Figure 6c,d).

**Figure 7:**
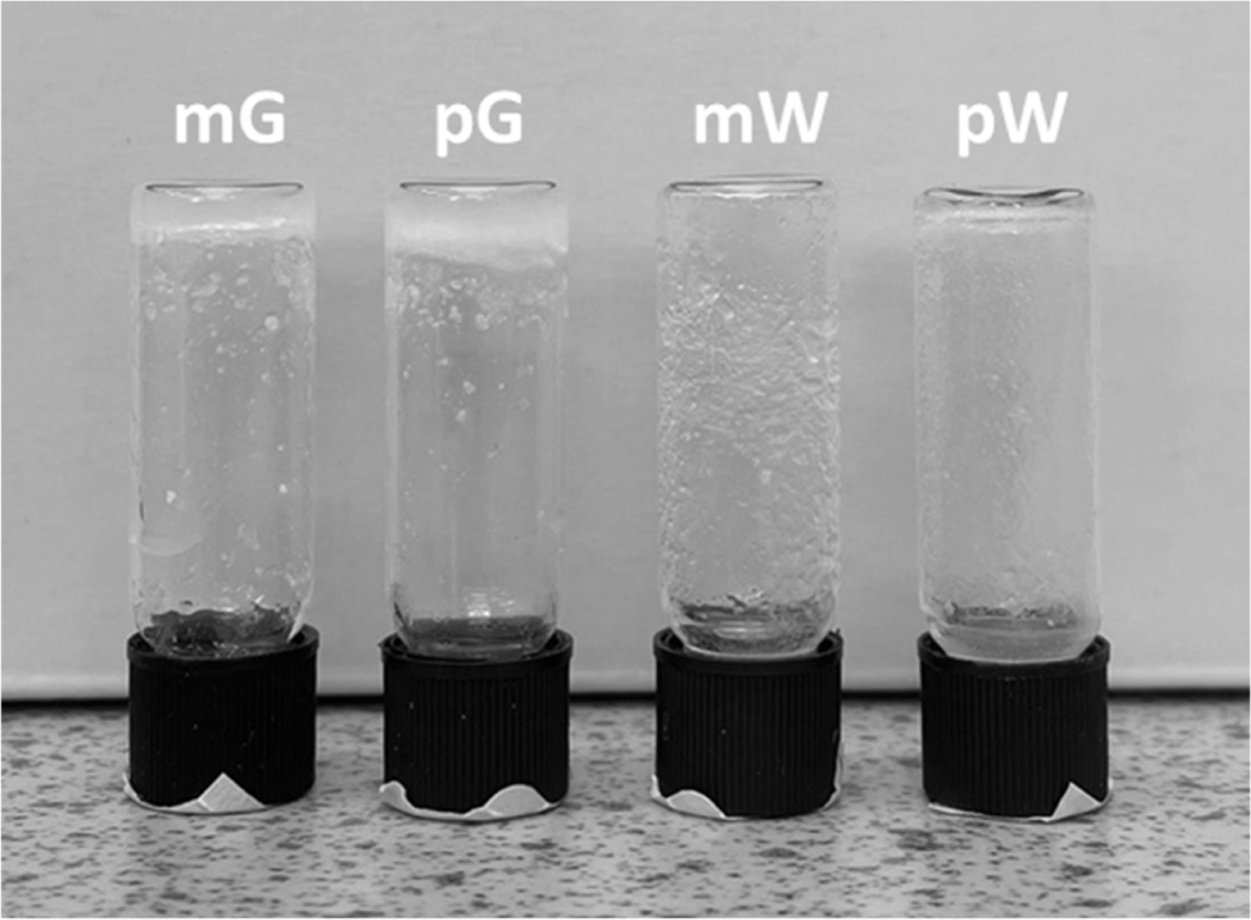
Tube inversion test showing hydrogel formation in 1 wt % PBS solutions of mG and pG.

The gelation capability of each peptide was tested at a concentration of 1 wt % in PBS. Samples were prepared by simply dissolving the peptide powder in PBS. After 10 min of sonication, as shown from an inverted test tube study (Figure 7), only mG and pG were able to form self-supporting hydrogels. This behavior can potentially be explained considering the less hydrophobic nature of the Gly residue compared to Trp. Moreover, a Gly residue has a lower steric hindrance compared to Trp, thus allowing a better packing of peptide side chains. These features could enable the formation of a more hydrophilic interface within the peptide network, with the capability to retain larger amounts of water.

Cryo-TEM images collected for the hydrogels of mG and pG are shown in Figure S6. These show that mG forms aligned straight nanotapes (apparently comprising parallel filaments as in the nanostructures in solution, Figure 4a) with a mean thickness of 13.6 nm. The hydrogel of pG contains twisted nanotapes (Figure S6b), again a structure similar to that observed in solution at 0.1 wt % (Figure 4b). The cryo-TEM images for the hydrogels thus confirm that these comprise similar nanotape structures to those formed in solution. This is also supported by the SAXS data for hydrogels shown in Figure S7a which have similar form factor profiles to those shown for the solution data in Figure 5.

Spontaneously formed hydrogels of 1 wt % mG and pG and 0.5 wt % mW and pW suspensions were stained with a Congo red solution and then visualized by optical microscopy (Figure S8). Under cross-polarized light, all of them exhibit characteristic green birefringence, suggesting an amyloid-like organization. SAXS data was measured for hydrogels of mW and pW (Figure S7b), and the form factor features are similar to those measured in solution, which indicates that the hydrogels and suspensions are formed from a network of entangled fibrils.

CD spectra collected for mG and pG hydrogels (Figure S9) have a similar shape to those for solutions above the CAC, with a positive band at 205 nm (Figure 1). By rotating the samples to four different positions, the CD profile was not found to differ (data not shown). This result excludes the presence of artifacts such as contributions from linear dichroism and confirms the homogeneity of the hydrogels.

In addition to spontaneous hydrogel formation at 1 wt % of mG and pG, it was possible to produce hydrogels after heat treatment (to 60 °C, followed by cooling to room temperature and leaving the sample for 24 h) for lower-concentration samples (0.5 wt %). Images of inverted tubes are shown in Figure S10, while Figures S11 and S12 show the corresponding FTIR and CD spectra. The inverted tube test showed that all four peptides form hydrogels, although the gel of pW was very soft and not self-supporting. The FTIR spectra measured for hydrogels formed after heating 0.5 wt % peptide solutions (Figure S11) reveal that a β-sheet organization is preserved for all the samples, as attested by the peaks at 1632 cm^–1^ for mG, 1629 cm^–1^ for pG, 1632 and 1624 cm^–1^ for mW, and 1636 and 1623 cm^–1^ for pW. The peaks at 1651, 1649, and 1652 cm^–1^ reveal a minor α-helix component for mG, mW, and pW, respectively. The peaks at 1647, 1643, and 1646 cm^–1^, observed for pG, mW, and pW, show that a minor component of unordered peptide is present. The CD spectra for the heat-treated hydrogels (Figure S12) also show similar features to those for the solutions and spontaneously formed hydrogels discussed above.

### Cytocompatibility

**Figure 8:**
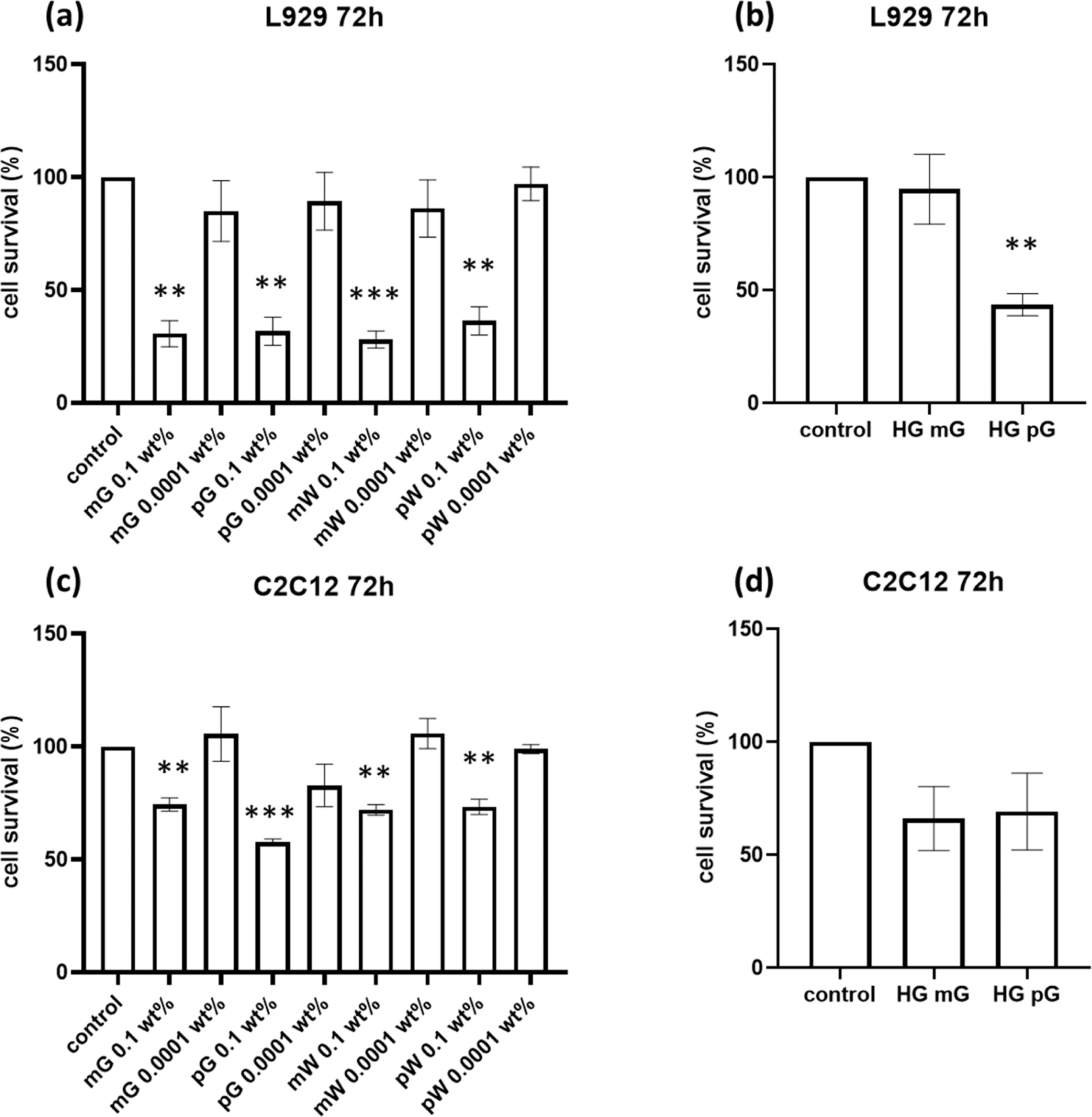
Cytotoxicity data from MTT assays obtained after 72 h. (a) L929 cells in solution, (b) L929 cells on hydrogels, (c) C2C12 cells in solution, and (d) C2C12 cells on hydrogels.

The cytotoxicity of peptides was assessed using MTT assays on L929 murine fibroblast and C2C12 immortalized mouse myoblast cell lines. The results obtained after 72 h of cell culture are shown in Figure 8. Additional data obtained after 24 h is shown in Figure S13. The *t*-test probability values are presented in Tables S3 and S4. The data in Figure 8a for L929 fibroblasts show that the cell viability is high (there was no significant difference when compared with the control groups) on all plates containing the lower lipopeptide concentration (1 × 10^–4^ wt %) solutions, with no significant difference from sample to sample. However, at 0.1 wt % (i.e., well above the CAC for all samples), the cell viability is significantly reduced to 30.7 ± 4.7%, 31.8 ± 5.1%, 28.1 ± 3.1%, and 36.3 ± 5.1% for mG, pG, mW, and pW respectively. These results suggest that self-assembled aggregates are not well tolerated, while monomers are. It is notable that the cytotoxicity of peptides at 0.1 wt % on L929 murine fibroblast is higher than that on C2C12 myoblasts. The cytotoxicity observed follows the trend observed after 24 h (Figure S13a), with significant cytotoxicity for the 0.1 wt % solution plates, with higher percentage cell viabilities for samples incubated with lower concentrations. The relative observed cytotoxicity was lower after 24 h incubation when compared with 72 h of incubation.

For L929 cells in contact with hydrogels (1 wt %), remarkably no significant cytotoxicity was observed for mG (cell survival of 92.6 ± 8.8% at 24 h and of 94.6 ± 12.6% at 72 h) (Figures 8b and S13b), but there was a notable reduction in cell viability for pG. Surprisingly, the cytotoxicity at this concentration is lower for both the peptides than for 0.1 wt % solutions, indicating that hydrogels are more cytocompatible than solutions, even with a higher lipopeptide content.

Cell viability measurements with C2C12 myoblasts revealed notable improvements in cytocompatibility at higher peptide concentrations as shown in Figure 8c. While the 0.1 wt % peptide-coated plates are somewhat cytotoxic, the cell viability is better than that observed for L929 fibroblasts by a factor of 2 or more, after 72 h. The cell viability was 84.4 ± 9.6%, 86.0 ± 6.8%, 72.3 ± 10.2%, and 73.3 ± 2.8% for mG, pG, mW, and pW, respectively. After 24 h, there is no significant cytotoxicity even for the higher concentration of lipopeptides (Figure S13c), with the exception of pW. The solutions prepared with 1 × 10^–4^ wt % lipopeptide showed minimal cytotoxicity, as for the L929 fibroblasts. Hydrogels were reasonably well tolerated by C2C12 cells (Figures 8d and S13d), with no substantial difference between mG and pG gels, in contrast to the superior cytocompatibility of mG gels with L929 fibroblasts.

## Conclusions

In summary, all four lipopeptides form extended β-sheet nanostructures at sufficiently high concentrations. The CAC was determined from CD measurements from discontinuities as a function of the concentration of the ellipticity of the positive band at 205 nm, associated with the β-sheet structure. For the two tryptophan-containing peptides, the CAC obtained from intrinsic W fluorescence was in good agreement with that determined by analysis of molar ellipticity. This was not the case using ThT dye due to interference between tryptophan and ThT fluorescence; however, the ThT fluorescence probe did give consistent CAC values for mG and pG. FTIR spectra reveal a predominant β-sheet structure for all four lipopeptides. Self-fluorescence due to amyloid aggregation was observed for mG and pG.

Cryo-TEM and SAXS were used to elucidate self-assembled nanostructures. Both mG and pG form twisted nanotape structures, the cryo-TEM images showing that these comprise individual filaments that aggregate side by side to form tapes. SAXS form factor data can be fitted using a bilayer form factor which represents the nanotape electron density profile by three Gaussian functions,^125^ one for the electron-poor hydrophobic lipid interior and the other two representing the hydrophilic and more electron-rich peptide surfaces. Unexpectedly and in contrast to the other lipopeptides, mW forms helical twisted ribbons which can be seen to close into nanotubes in clearly resolved cryo-TEM images. Oscillations in the SAXS form factor were used to determine the average nanotube radius, which was in good agreement with that obtained from the cryo-TEM images. Lipopeptide pW forms a dense network of fibrils with a different morphology to those of mG and pG, although the SAXS data could still be fitted using a bilayer form factor model. Both mW and pW show birefringence due to lyotropic nematic phase formation and spontaneous flow alignment of this phase upon delivery into X-ray capillaries was noted. Nematic phase formation is relatively infrequently observed for amyloid systems^126−130^ and points to the high persistence length of the nanotube, twisted ribbon, and nanotape structures of mW and pW.

The distinct structures formed by mW and pW highlight the effect of the bulky and chiral tryptophan residue compared to glycine in the nanostructure formation of the four lipopeptides. The tryptophan residue is likely to lead to a locally more chiral and twisted packing, but this is modulated by alkyl chain length. The highest degree of twist of β-sheet structures is observed for mW, whereas pW appears to form thinner and more extended but less twisted structures. Therefore, both the N-terminal residue and the alkyl chain length influence molecular packing and self-assembly. Even a small difference of two methyl groups in the lipid chain substantially influences aggregation. Our work shows that the incorporation of tryptophan residues at the lipid–peptide junction can be used to tune self-assembly, as well as providing a useful fluorescence tag for aggregation studies.

The cell assays indicate that all lipopeptides show good cytocompatibility at lower lipopeptide concentrations (below the CAC). In addition, there are only minor differences from sample to sample. This is despite differences in self-assembled nanostructure noted above, in particular the tendency for mW to form nanotubes. Another notable trend was the generally greater tolerance for hydrogels than solution coatings, even though the former were prepared with a higher lipopeptide concentration. One reason for this may be the contact surface between the peptides and hydrogels. The cells seeded on the hydrogels are predominantly located on the surface, whereas the ones incubated with liquid solutions are totally enveloped by the medium containing lipopeptide. The content of free lipopeptide in the medium may differ between hydrogels and solutions. Cell viability data reveal a significant difference in the tolerance of lipopeptide coatings for C2C12 myoblasts compared to L929 fibroblasts at higher concentrations (0.1 wt %, well above the determined CAC values) for all lipopeptides. This selective improvement in cytocompatibility is promising for future applications of self-assembled (and monomeric) lipopeptide coatings in muscular tissue engineering, especially relevant to the production of cultured meat. It is not uncommon to observe multiple responses for the same compound between different cell lines, be it related to metabolic activity, cytotoxicity, proliferation, or cell adhesion. This effect was also observed previously in other RGDS-based molecules, with different levels of cell adhesion and cytotoxicity for the same molecule for distinct cell types.^34,231,232^

A possible explanation for the increased cytotoxicity in fibroblasts is the fact that soluble RGD-peptides may trigger apoptosis by the inhibition of vitronectin and fibronectin domains, promoting cell detachment and leading to anoikis, a type of programmed cell death that occurs in anchorage-dependent cells upon detachment from the surrounding extracellular matrix or substrate, an effect that has already been observed for fibroblasts.^32,132,133^

The observation of fibril textures for the lipopeptides (which in several cases show spontaneous flow alignment) is particularly interesting in terms of further research underway on the preparation of aligned scaffolds for tissue engineering^131^ using the alignment of myoblasts to improve the texture of cultured meat.

## Supporting Information Available

HPLC data, SAXS data fit parameters, fluorescence data, FTIR spectra, additional cryo-TEM images, SAXS and CD data for hydrogels, polarized optical microscopy images, images of heat-treated hydrogels, and cytotoxicity data from MTT assays obtained after 24 h (PDF)
