# Supplementary material for: Cell Adhesion Motif-Functionalized Lipopeptides: Nanostructure and Selective Myoblast Cytocompatibility
Source: Biomacromolecules. 2022 Dec 15;24(1):213–24. doi: 10.1021/acs.biomac.2c01068 (PMC9832505; doi:10.1021/acs.biomac.2c01068)
Supplement: Supplementary file 1 — bm2c01068_si_001.pdf [file bm2c01068_si_001.pdf]

Supporting Information

Cell Adhesion Motif Functionalized Lipopeptides: Nanostructure and Selective Myoblast Cytocompatibility

Elisabetta Rosa,[a,b] Lucas de Mello,[a,c] Valeria Castelletto,[a] Mark L. Dallas,[a] Antonella Accardo,[b] and Ian W Hamley[a,]*

[a] *School of Chemistry, Pharmacy and Food Biosciences, University of Reading, Whiteknights, Reading, Berkshire RG6 6AD, U.K.*

[b] *Department of Pharmacy and Research Centre on Bioactive Peptides (CIRPeB), University of Naples "Federico II", Via Domenico Montesano 49, Naples, 80131, Italy*

[c] *Departamento de Biofísica, Universidade Federal de São Paulo, São Paulo 04023-062, Brazil*

* I.W.Hamley@reading.ac.uk. ORCID ID: 0000-0002-4549-0926

Tables

Table S1. Parameters extracted from the fitting of the SAXS data[a].

	**mG** **0.5 wt%**	**pG** **0.1 wt%**	**mW** **0.1 wt%**	**pW** **0.5 wt%**
$t \pm \Delta t$ [Å]	46.2 ± 5.0	39.0 ± 7.6		35.0 ± 3.0
η_{out}	3.3×10^{-6}	6.0×10^{-7}		3.0×10^{-6}
σ_{out} [Å]	10.6	12.3		10.0 [b]
η_{in}	-1.1×10^{-6}	-4.2×10^{-7}		-5.0×10^{-7}
σ_{in} [Å]	29.4	18.0		10.0[b]
D [Å]	745	2103		500[b]
R [Å]			751 ± 200	
ΔR [Å]			64	
η_{core}			1×10^{-7} [b]	
η_{shell}			2.3×10^{-7}	
η_{solv}			1×10^{-7} [b]	
L			2000[b]	
C	0.001[b]	0[b]	0.01[b]	0.1[b]

[a] Data fitted using Gaussian bilayer for nanotapes (mG, pG and pW) and long core-shell cylinder for nanotubes (mW) using the software SASfit.[1]

[b] Fixed parameter

Key: Gaussian bilayer: layer thickness t (Gaussian polydispersity Δt), scattering contrast of outer layers η_{out}, and inner layer η_{in}, Gaussian widths σ_{in} and σ_{out} of inner and outer layers respectively, D diameter (width) of layer system (when $D >> t$ as here, it acts as a scaling parameter for the form factor). **Nanotubes**: R core radius, ΔR shell thickness, scattering contrasts of core η_{core}, shell η_{shell} and solvent η_{solv}, L length. **Background:** constant background, C.

Table S2. CAC values (all wt%) calculated using different techniques.

	CD	ThT fluorescence	W fluorescence
mG	$(6.02 \pm 0.03)\times10^{-3}$	$(4.59\pm 0.05)\times10^{-3}$	
pG	$(6.49 \pm 0.08)\times10^{-3}$	$(2.87 \pm 0.04)\times10^{-3}$	
mW	$(2.16 \pm 0.05)\times 10^{-4}$	$(3.10 \pm 0.07)\times10^{-3}$	$(2.16\pm0.05)\times10^{-4}$
pW	$(1.26 \pm 0.03)\times10^{-4}$	$(3.55\pm 0.08)\times10^{-3}$	$(3.75\pm 0.04)\times10^{-4}$

Table S3. Statistical significance *t*-test p values for L929 and C2C12 cells after 24h and 72h.

System	p value / L929 24h	p Value / L929 72h	p Value / C2C12 24h	p Value / C2C12 72h
control vs. mG 0.1 wt%	0.0065	0.0023	0.1490	0.0045
control vs. mG 0.0001 wt%	0.0957	0.1932	0.0588	0.4702
control vs. pG 0.1 wt%	0.0042	0.0027	0.1023	0.0004
control vs. pG 0.0001 wt%	0.0522	0.2845	0.0716	0.0874
control vs. mW 0.1 wt%	0.0054	0.0009	0.0617	0.0024
control vs. mW 0.0001 wt%	0.0216	0.1973	0.2534	0.2741
control vs. pW 0.1 wt%	0.0068	0.0032	0.0167	0.0053
control vs. pW 0.0001 wt%	0.5100	0.5647	0.1497	0.4295

Table S4. Statistical significance *t*-test p values for L929 and C2C12 cells on hydrogels after 24 h and 72h

System	p Value / L929 24h	p Value / L929 72h	p Value / C2C12 24h	p Value / C2C12 72h
control vs. HG mG	0.3593	0.6118	0.1915	0.0533
control vs. HG pG	0.0406	0.0026	0.1658	0.0881

Figures

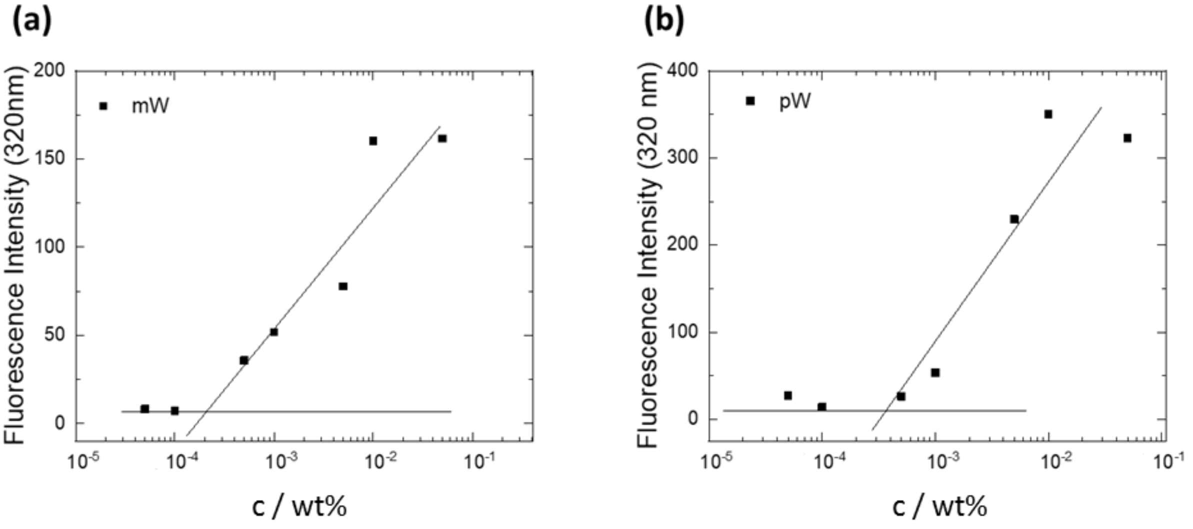

Fig.S1. Concentration-dependent W fluorescence measurements to determine CAC values (from intersection of straight lines shown). (a) mW, (b) pW.

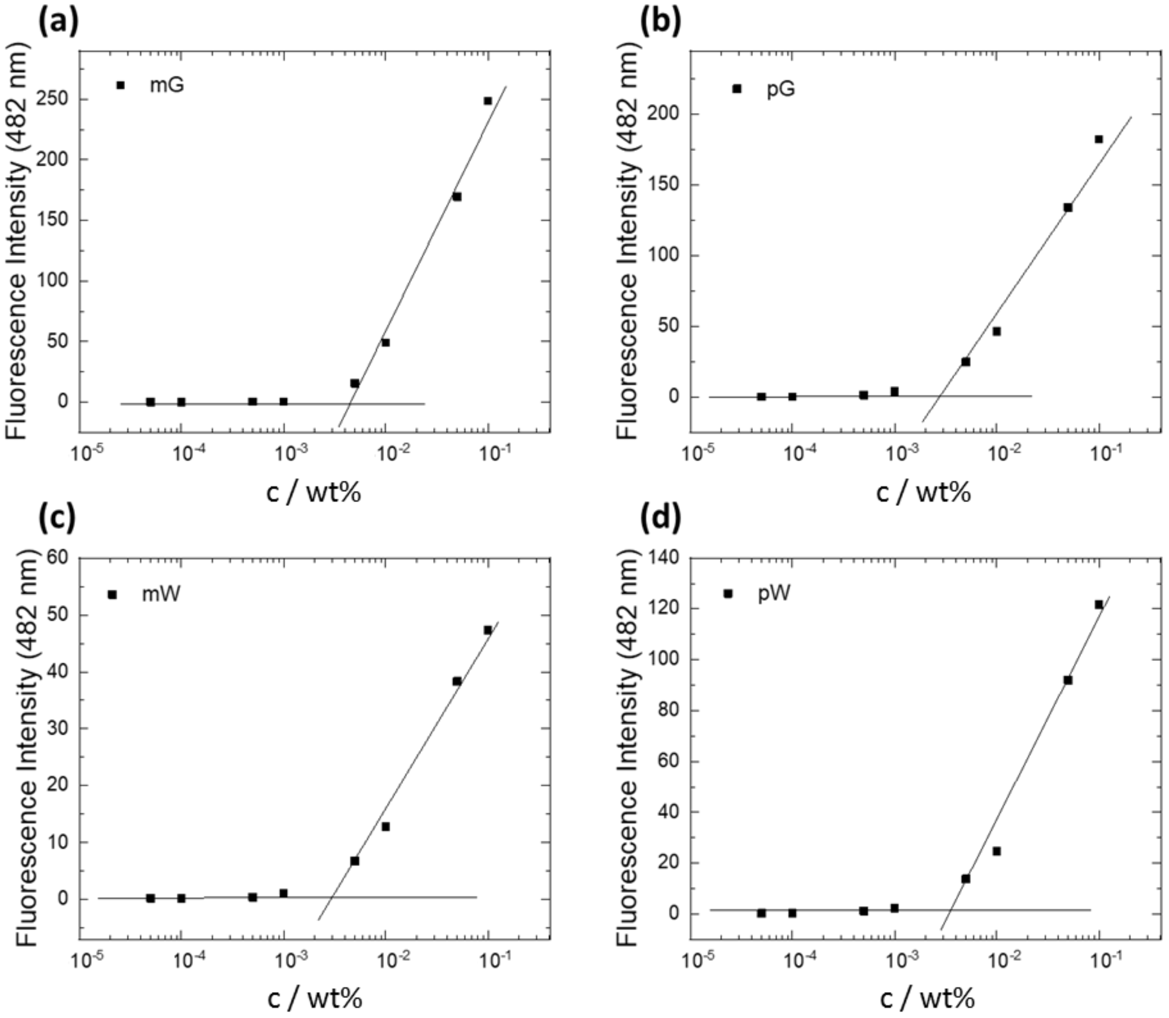

Fig.S2. Concentration-dependent ThT fluorescence measurements to determine CAC values (from intersection of straight lines shown). (a) mG, (b) pG, (c) mW, (d) pW.

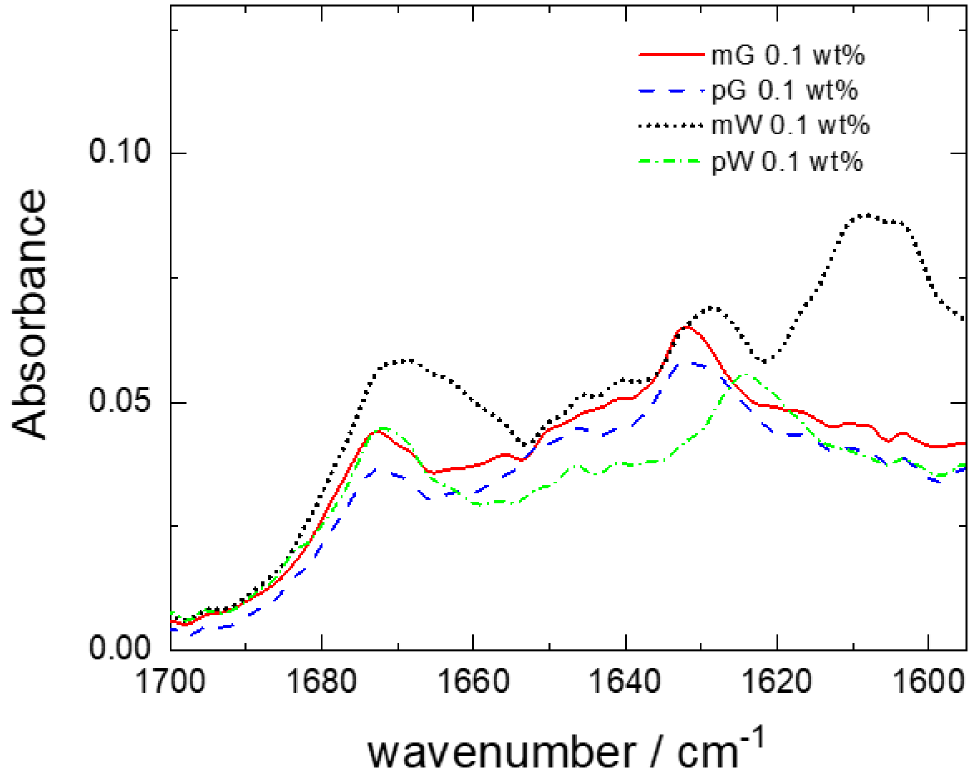

Fig.S3. FTIR spectra for 0.1 wt% samples

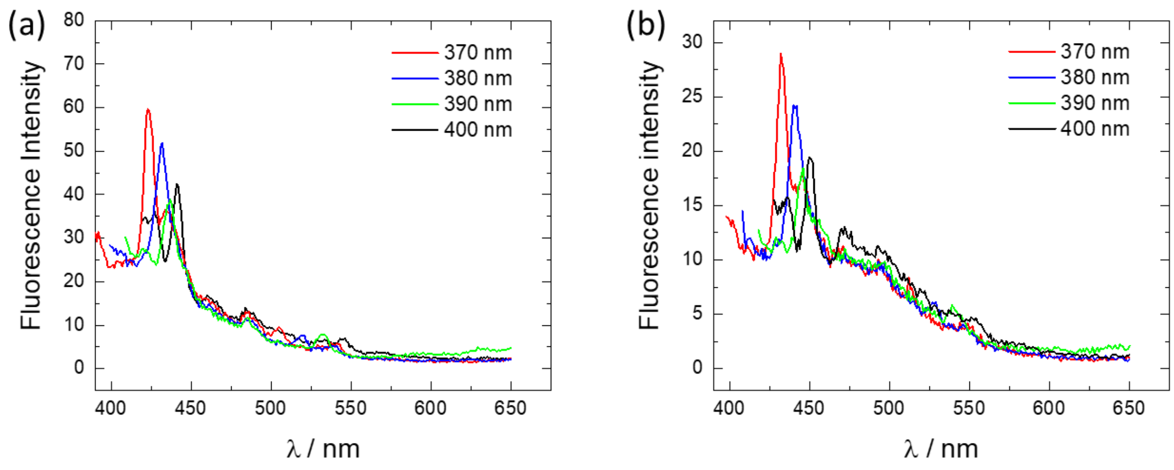

Fig.S4. Self-fluorescence of W-containing lipopeptides. (a) mG, (b) mW.

(a) mG

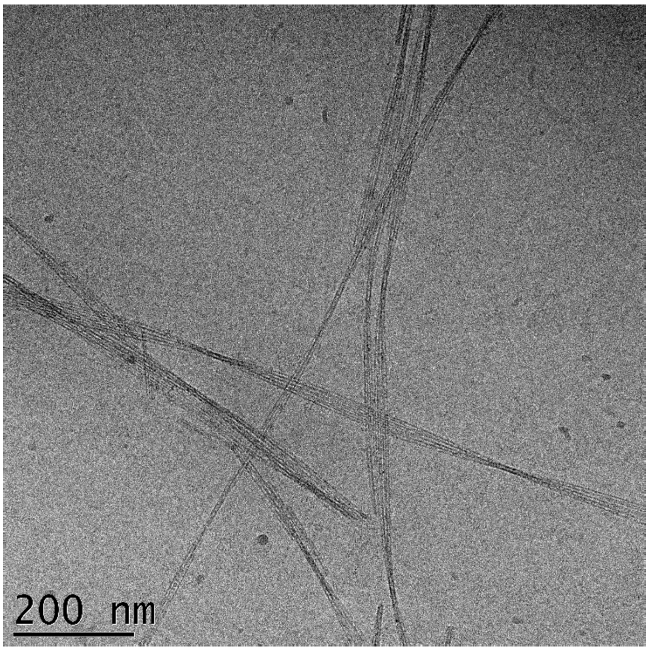

(b) pG

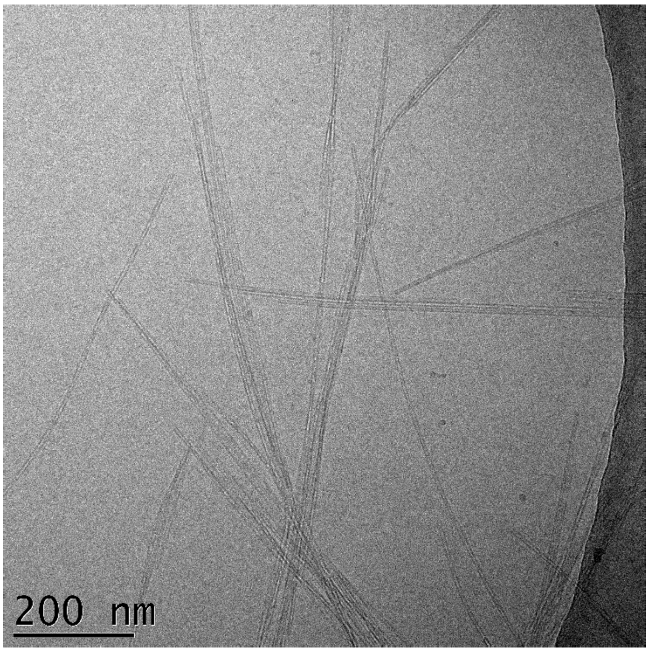

(c) mW

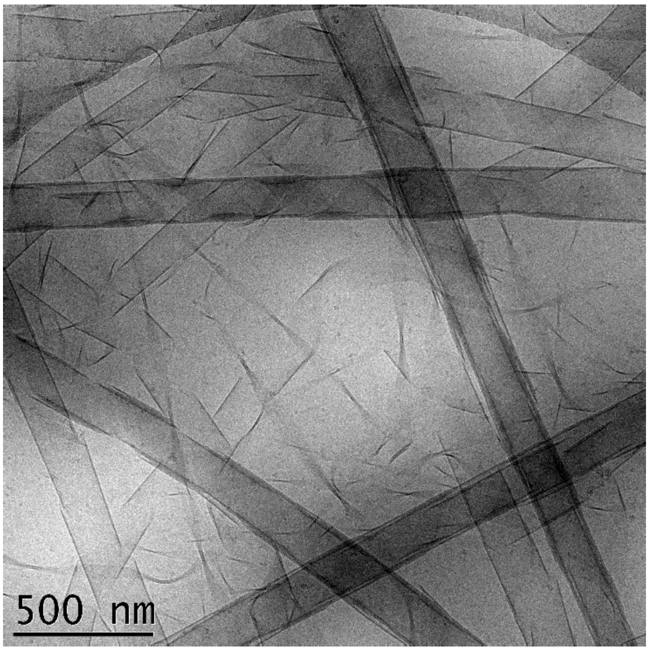

(d) pW

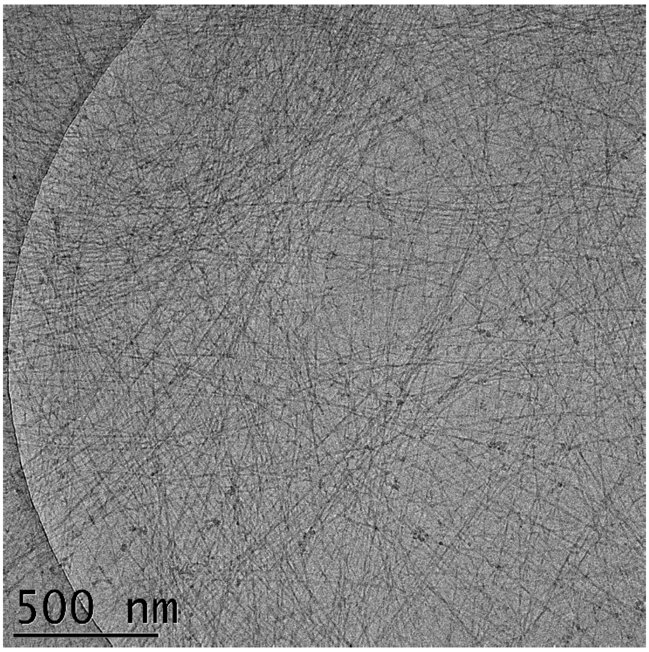

Figure S5. Additional cryo-TEM images from 0.1 wt% solutions of (a) mG, (b) pG, (c) mW, (d) pW.

(a) mG

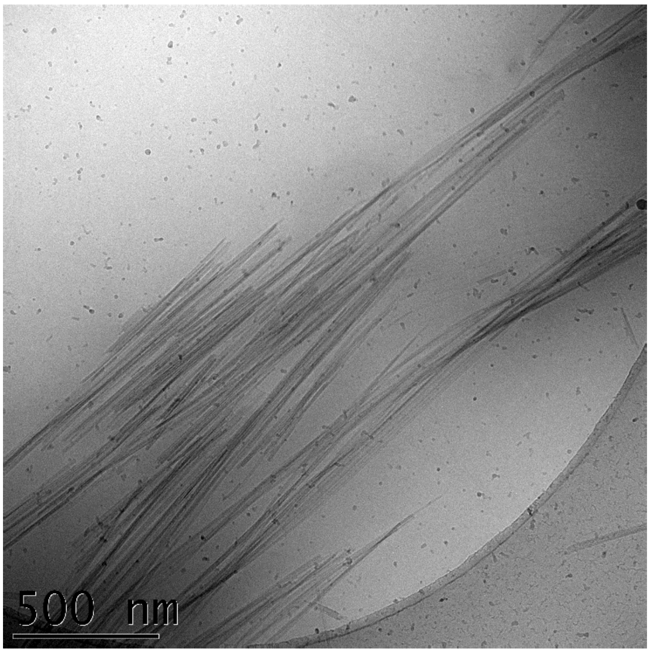

(b) pG

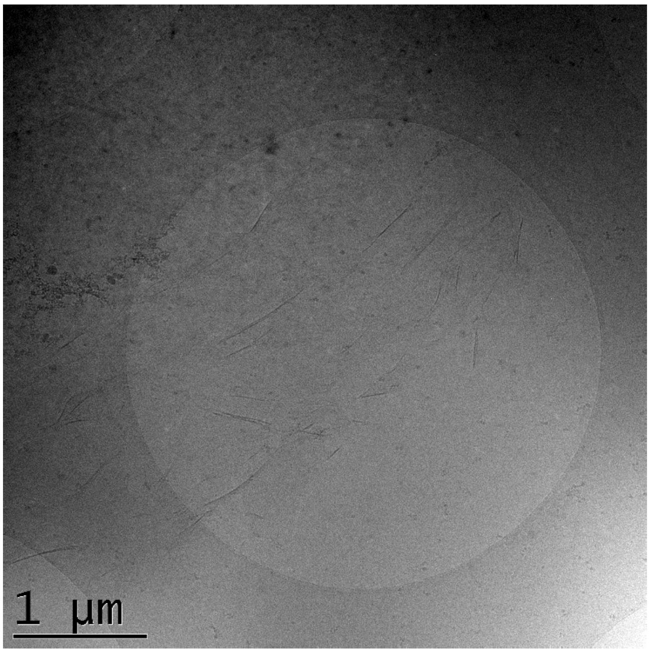

Fig.S6. Cryo-TEM images from 1 wt% hydrogels of (a) mG, (b) pG.

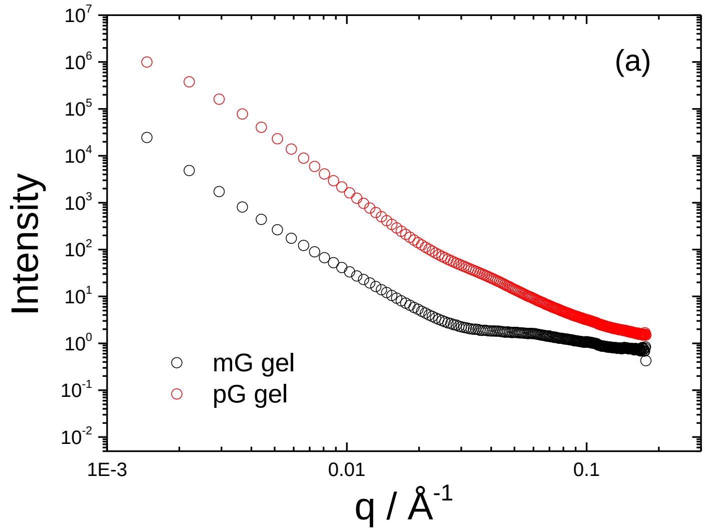

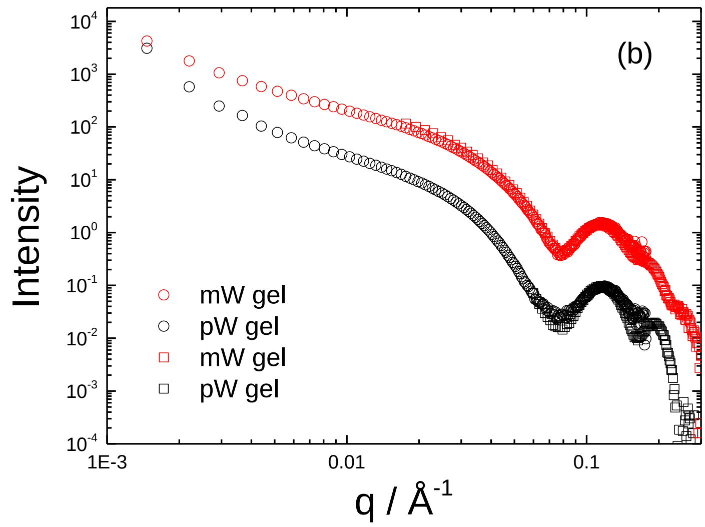

Fig.S7. SAXS intensity profiles from gelled 1 wt% samples of (a) mG and pG (single *q* range, no background subtraction). (b) mW and pW. Data were measured over two *q* ranges shown with two symbols, profiles were manually overlapped. The data for pW has been scaled down by a factor of 10.

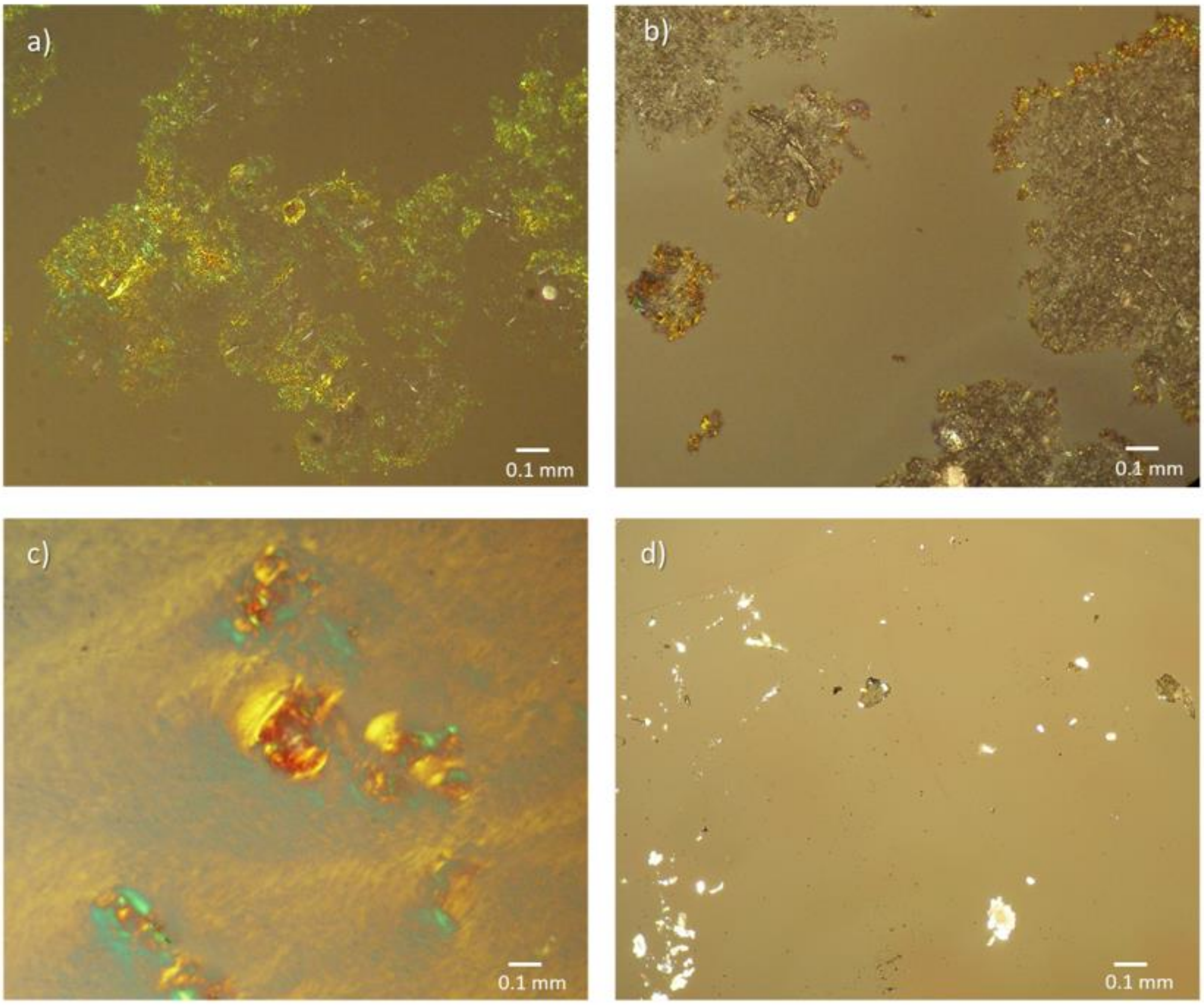

Fig.S8. Polarized optical microscopy images of 1 wt% samples stained with Congo red, (a) mG, (b) pG, (c) mW, (d) pW.

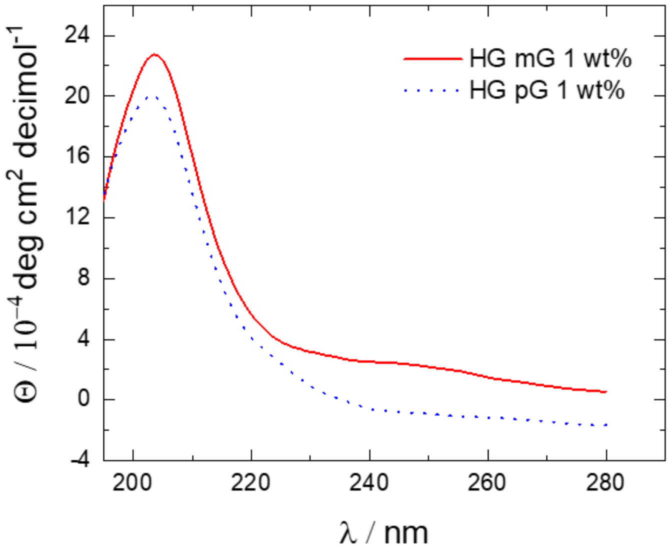

Fig.S9. CD spectra for mG and pG hydrogels (1 wt%).

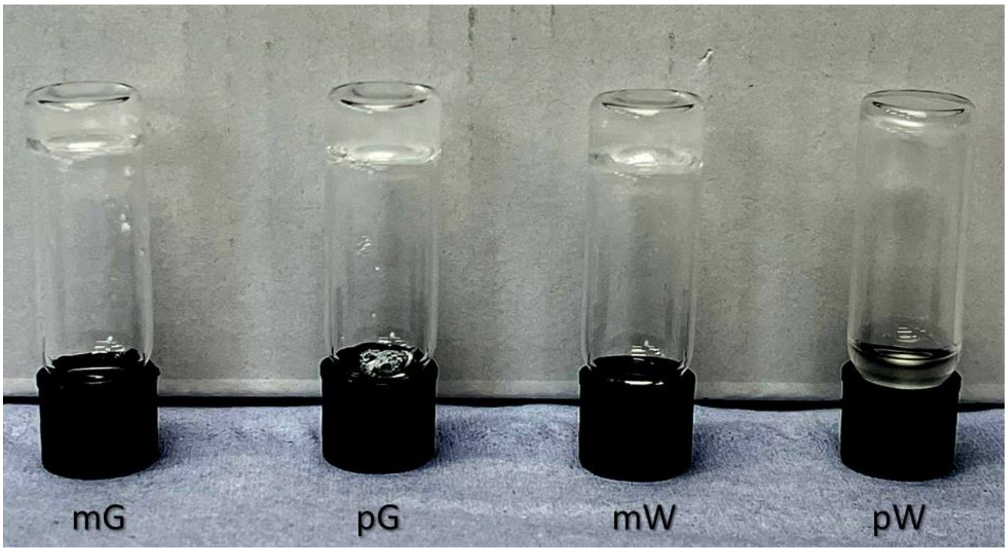

Fig.S10. Images of inverted tubes containing heat-treated hydrogels (0.5 wt%) as indicated. Sample pW formed a soft hydrogel at the base of the tube.

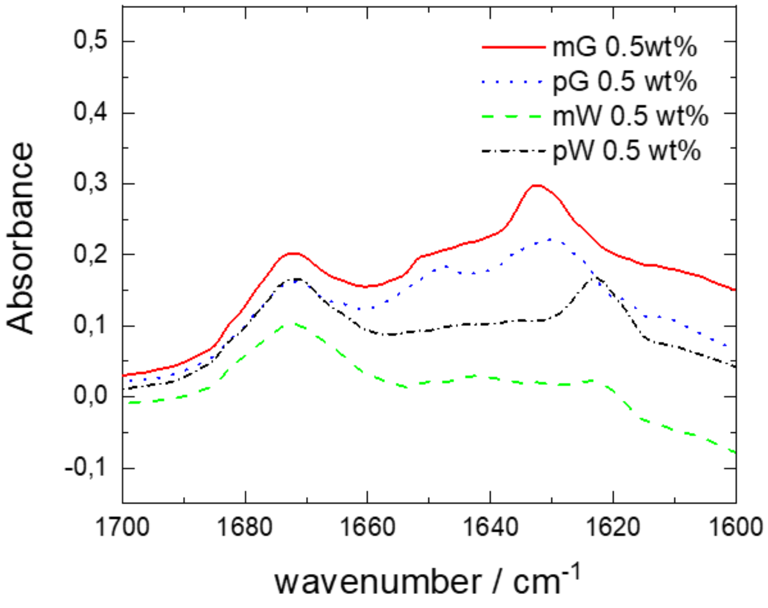

Fig.S11. FTIR spectra for heat-treated hydrogels (0.5 wt%).

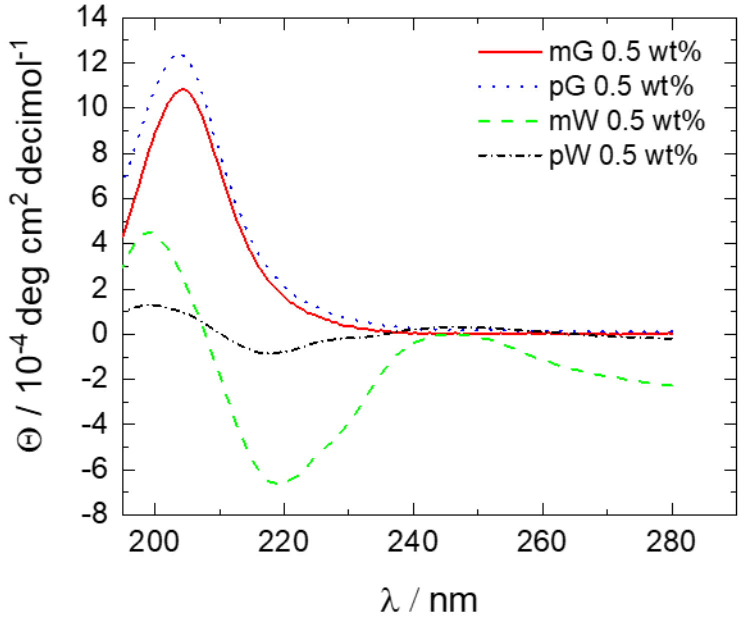

Fig.S12. CD spectra for heat-treated hydrogels (0.5 wt%).

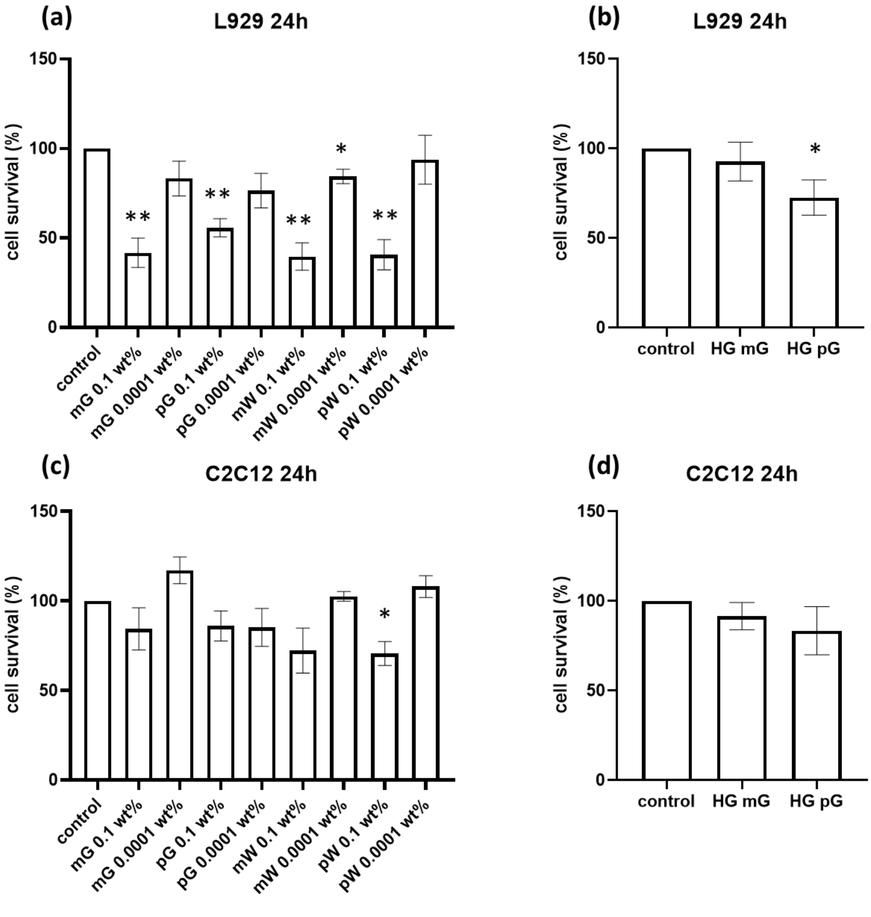

Fig.S13. Cytotoxicity data from MTT assays obtained after 24 h. (a) L929 cells in solution, (b) L929 cells on hydrogels (hg), (c) C2C12 cells in solution, (d) C2C12 cells on hydrogels.

Reference

(1) Bressler, I.; Kohlbrecher, J.; Thünemann, A. F., SASfit: a tool for small-angle scattering data analysis using a library of analytical expressions. *J. Appl. Crystallogr.* **2015,** *48*, 1587-1598.
